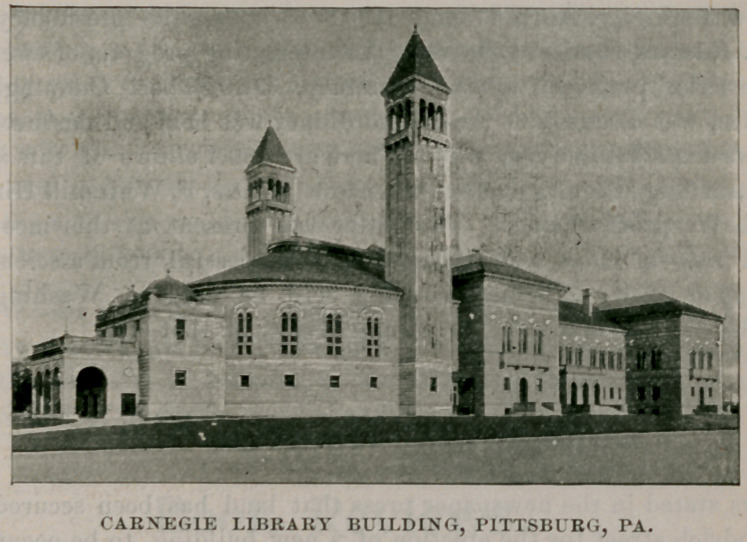# Society Meetings

**Published:** 1896-05

**Authors:** 


### Society Meetings.

### THE ATLANTA MEETINGS.

A large group of medical meetings will be held at Atlanta, Ga., during the first week in May. The following is the list in the order of their appointed meetings: American Academy of Medicine, May 2d and 4th, at the Hotel Aragon; Medical Publishers' Association, 9.30 A. M., May 4, room 105, Kimball House; National Confederation of State Medical Examining and Licensing Boards, Monday, May 4th, 10 o'clock A. M., breakfast room, Hotel Aragon; Georgia Pharmaceutical Association, Monday, May 4th, hour and place to be named ; Southern Railroad and Alabama Great Southern Railroad Surgeons, in the ball room of the Kimball House, Monday, May 4th, at an hour to be named ; the American Editors Association, in the Kimball House banquet hall, Monday evening, May 4th, 7 o'clock ; the American Medical Association, at the Grand, Tuesday, May 5th, at 11 o'clock A. M., to continue four days.

Dr. Willis F. Westmoreland is chairman of the committee of arrangements on the part of the American Medical Association and Dr. J. McFadden Gaston is chairman of the committee of arrangements on the part of the American Academy of Medicine. To the untiring efforts of these two men is due the admirable arranging and placing of this large group of meetings.

---

THE American Orthopedic Association will hold its tenth annual meeting at Buffalo, Tuesday, Wednesday and Thursday, May 19, 20 and 21, 1896, under the presidency of Dr. Royal Whitman, of New York. The secretary, Dr. John Ridlon, of Chicago, has prepared the following preliminary program :

The president's address, by Dr. Royal Whitman, New York. Some practical points in the treatment of lateral curvature of the spine, by Dr. A. B. Judson, New York. Some etiological factors in lateral curvature of the spine, by Dr. E. G. Brackett, Boston. Case illustrating the absurdity of treating ordinary lateral curvature (scoliosis) by spinal supports, by Bernard Roth, F. R. C. S., London. The rationale of gymnastic exercise and pressure correction in the treatment of scoliosis, by Dr. L. A. Weigel, Rochester. The rapid cure of rotary lateral curvature of the spine and other postural deformities, by means of thorough development and corrective exercises with heavy weights, with a demonstration of the method, by Dr. Jacob Teschner, New York (by invitation). A simple and efficient brace for lateral curvature, by Dr. S. L. McCurdy, Pittsburg. Congenital misplacement of the femur anteriorly, by Dr. DeForest Willard, Philadelphia. Further remarks on congenital dislocation of the hip, by Bernard E. Brodhurst, F. R. C. S., London. Report of a case of double congenital dislocation of the hip, treated by the Lorenz method of operation, by Dr. Reginald H. Sayre, of New York. The cure of congenital dislocation of the hip by means of the "functional weighting" method, without open operation, by Dr. Adolf Lorenz,

Vienna. Spontaneous dislocation of the hip, by Dr. William J. Taylor, Philadelphia. The treatment of club-foot: (*a*) when to commence treatment and how; (*b*) the indications for mechanical treatment; (*c*) the limitations of mechanical treatment; (*d*) the indications for operative treatment; (*e*) results in 343 operations performed by the writer, by Dr. A. M. Phelps, New York. Investigations on flat-foot, by Dr. E. H. Bradford, Boston. Mechanical support for flat-foot, by Dr. John C. Schapps, Brooklyn. The anterior transverse arch of the foot, by Dr. Joel E. Goldthwait, Boston. Injuries of the tarsus and the ankle joint, by Dr. J. D. Griffith, Kansas City. Subtendinous exostosis, by Dr. E. G. Brackett, Boston. The mechanical treatment of ingrown toe nail, by Dr. Henry Ling Taylor, New York. The operative treatment of paralytic deformities of the foot, with particular reference to arthrodesis, by Dr. V. P. Gibney, New York. Some mechanical problems in the treatment of Pott's disease, by Dr. John C. Schapps, Brooklyn. The operative treatment of threatening abscesses in the high dorsal region, by Dr. E. H. Bradford, Boston. The treatment of Pott's paraplegia, with a report of two cases, by Dr. LeRoy W. Hubbard, New York. Osteomyelitis of the spine, by Dr. T. Halsted Myers, New York. Suppuration in joint and spinal disease and its relation to tubercular meningitis; an analytical study, by Dr. Samuel Ketch, New York. A study of the action of iodoform glycerine in tubercular osteomyelitis, by Dr. Harry M. Sherman, San Francisco. Joint disease in infancy, by Dr. Augustus Thorndike, Boston. The use of dry heat of high temperature in the treatment of chronic joint affections, by Dr. William E. Wirt, Cleveland. A theory of the ultimate etiology of deformity and its practical application, by Dr. Royal Whitman, New York. The probable cause of the limp in the first and second stage of hip-joint disease, by Dr. Harry M. Sherman, San Francisco. Femoral osteotomy for correction of hip deformity in adults, with a report of cases, by Dr. A. R. Shands, Washington (by invitation). A report of cases of osteosarcoma of the hip, by Dr. Arthur J. Gillette, St. Paul. Division of the hamstring tendons by the open method for correcting malposition and securing rest in tubercular disease of the knee, by Dr. Bernard Bartow, Buffalo. Tuberculosis of the wrist and carpus, by Dr. James E. Moore, Minneapolis. Symptoms and treatment of slight knock-knee in children, by Dr. Robert W. Lovett, Boston. Two cases of dislocation of the patella treated by operation, by Dr. Joel E. Goldthwait, Boston. Some notes on spastic paralysis in children, by Dr. F. S. Coolidge, Chicago. Some recent modifications in the treatment of congenital wry neck, by William Adams, F. R. C. S., London. Contracted fingers, by Dr. Arthur J. Gillette, St. Paul. Congenital club-hand, the report of a case treated by operation, by Dr. C. E. Thomson, Scranton (by invitation). Rare cases from practice, by Dr. A. J. Steele, St. Louis. A report of some cases of unusual congenital deformities, by Dr. John Ridlon, Chicago. Congenital defects of the long bones, a report of cases and operations, by Dr. B. E. McKenzie, Toronto. Deformities of the humerus due to rickets, by Dr. Augustus Thorndike, Boston. A report of a family of anomalies, by Dr. S. L. McCurdy, Pittsburg.

In addition to the scientific program there will be a number of social functions, including an excursion to Niagara Falls, all under the management of Drs. Park and Bartow, of Buffalo, as a committee of arrangements.

The meetings of the association will be held at Alumni Hall, in the University of Buffalo and a cordial invitation is extended to all physicians to attend the several scientific sessions.

---

The Alumni Association of the medical department of the University of Buffalo will hold its twenty-first annual meeting in connection with the semi-centennial anniversary of the establishment of the University, Tuesday, May 5, 1896, at Alumni Hall, University building. Following is the program:

Morning Session, 10.30 A. M.—Registration, report of executive committee, revision of constitution and by-laws, general report, report of treasurer, new business, election of officers for 1896–97.

Afternoon Session, 2 P. M.—(The discussions during the afternoon session will be limited to ten minutes.) President's address, Willis M. Baker, M. D. Colpoperineorrhaphy, Henry J. Garrigues, M. D., New York City, N. Y.; discussion, Drs. Roswell Park, M. A. Crockett, Eugene A. Smith. The state of the gastric mucosa in secretory disorders of the stomach, Max Einhorn, M. D., New York City, N. Y.; discussion, Drs. Charles G. Stockton, Allen A. Jones, A. L. Benedict. Reconstructive surgery of the tubes and ovaries, Robert T. Morris, M. D., New York City, N. Y.; discussion, Drs. M. D. Mann, W. W. Potter, C. C. Frederick. Some notes on the coronary arteries, George Dock, M. D.,

Ann Arbor, Mich.; discussion, Drs. H. R. Hopkins, Chas. Cary, DeLancey Rochester.

Commencement exercises, Music Hall, 7.30 P. M. Banquet, Hotel Genesee, 9.30 P. M.

The graduation exercises will be held in Music Hall at 7.30 o'clock in the evening, and members and their friends are invited to attend. Charles O'Connor will address the graduating class.

This year the annual dinner will be given at the Hotel Genesee, and seats will be taken as near 9.30 o'clock as possible. Tickets will be on sale ($2.00) during the morning and afternoon sessions, and also at the Hotel Genesee.

The executive committee again urges all alumni to enroll themselves as active members of the association. The remarkable advancement and success of the university is a benefit to each alumnus, and he should still further show his loyalty to his alma mater by sustaining the good work with his attendance, efforts and contributions.

The following named are the present officers of the association: President, Willis M. Baker, Warren, Pa.; first vice-president, P. W. Van Peyma, Buffalo, N. Y.; second vice-president, D. A. Currie, Englewood, N. J.; third vice-president, Herman G. Matzinger, Buffalo, N. Y.; fourth vice-president, J. A. McPherson, Tonawanda, N. Y.; fifth vice-president, J. W. Putnam, Buffalo, N. Y.; permanent secretary, E. L. Frost, Buffalo, N. Y.; recording secretary, N. V. Chappell, Buffalo, N. Y.; treasurer, H. U. Williams, Buffalo, N. Y.

Board of trustees: F. E. L. Brecht, 1896, Buffalo, N. Y.; E. C. W. O'Brien, 1897, Buffalo, N. Y.; Jos. Fowler, 1898, Buffalo, N. Y.; Julius Wenz, 1899, Lancaster, N. Y.; H. P. Trull, 1900, Williamsville, N. Y.

Executive committee: Allen A. Jones, chairman, Buffalo, N. Y.; Albert T. Lytle, secretary, Buffalo, N. Y.; H. G. Matzinger, Buffalo, N. Y.; W. M. Baker, president, *ex-officio;* John Parmenter, secretary of the faculty, *ex-officio.*

---

THE Ohio State Pediatric Society will hold its annual meeting at Columbus, on Wednesday, May 27, 1896. Those who have papers to present should at once communicate with the secretary, Dr. Geo. M. Clouse, of Columbus, giving title of paper. The other officers of the society are: president, Dr. S. W. Kelly, Cleveland; vice-president, Dr. J. P. West, Bellaire; chairman of council, Dr.

J. M. Dunham, of Columbus. Any regular physician who is particularly interested in pediatrics and a worker therein is eligible to become a member of this young and growing society. This is the first pediatric society to organise within state boundary lines. The diseases of children are of increasing importance and a full attendance at this meeting is particularly urged.

---

THE American Microscopical Society will hold its nineteenth annual meeting in the new Carnegie Library Building, Pittsburg, Pa., Tuesday, Wednesday, Thursday and Friday, August 18, 19, 20 and 21, 1896. A hearty welcome will be extended to all inter-

CARNEGIE LIBRARY BUILDING, PITTSBURG, PA.

ested in the microscopical sciences. Applications for membership and titles of papers to be read at the meeting should be addressed to A. Clifford Mercer, M. D., president, Syracuse, N. Y., or to Wm. C. Krauss, M. D., secretary, 382 Virginia street, Buffalo, N. Y.

---

THE Association of Military Surgeons of the United States will hold its sixth annual meeting at Philadelphia, Tuesday, Wednesday and Thursday, May 12, 13 and 14, 1896, under the presidency of Col. Louis W. Read, M. D., Surgeon-General of Pennsylvania, Norristown. The secretary, Lieut.-Col. Eustathius Chancellor, Medical Director N. G. Mo., of St. Louis, has sent out an elaborate program, consisting of twenty-seven titles.

Medical Director Albert L. Gihon, U. S. Navy (retired), is first vice-president, and Major Albert H. Briggs, M. D., N. G. N. Y., Buffalo, is a member of the executive council.

The morning session of Tuesday will be held at the Broad street theater at 10 o'clock, where addresses will be made by Governor Hastings and others, while the regular business and scientific sessions will be held at the Hotel Walton. There will be numerous social functions and it is expected that this meeting will be one of the largest and best in the history of the association.

---

The American Laryngological, Rhinological and Otological Society held its second annual meeting at the Academy of Medicine, New York city, April 17 and 18, 1896, under the presidency of Dr. Edward Bradford Dench. An interesting program of twenty-five titles, prepared by the secretary, Dr. Robert Cunningham Miles, was discussed. An annual dinner was held and the meeting was successful in every way. There are 108 Fellows of this society, among whom we notice the names of Drs. F. Whitehill Hinkel and W. Scott Renner. The latter was present at this meeting and reports it as well attended and successful from a scientific point of view. The next meeting will be held in Washington under the presidency of Dr. Frank Hyatt, of that city.

## Figures and Tables

**Figure f1:**